# Short-Term Outcomes After Corticosteroid Injection in Patients with Clinically Diagnosed Subacromial Pain Suggestive of Bursitis

**DOI:** 10.3390/jcm15207863

**Published:** 2026-10-12

**Authors:** Christoph A. Schroen, Alexander Yu, Akiro H. Duey, Sri Guttikonda, Michael Li, Richard Hausman, Jordan Bernstein, William A. Ranson, Michael R. Hausman, Paul J. Cagle

**Affiliations:** 1 Department of Orthopaedic Surgery, Icahn School of Medicine, Mount Sinai, 1468 Madison Avenue, New York, NY 10029, USA; alexander.yu@icahn.mssm.edu (A.Y.); william.ranson@mountsinai.org (W.A.R.);; 2 Medical Faculty Heidelberg, Heidelberg University, 69120 Heidelberg, Germany

**Keywords:** subacromial bursitis, shoulder, corticosteroid injection, shoulder pain, range of motion, bursitis

## Abstract

**Background:** The current diagnostic paradigm for clinically suspected subacromial bursitis is based on clinical history and physical examination. However, clinical signs frequently overlap with other pathologies. This study investigated short-term outcomes following the currently employed clinical criteria by comparing patients who met these criteria and received an injection with those who were offered but refused an injection. **Methods:** From July 2024 to April 2025, patients with symptoms of clinically suspected subacromial bursitis were enrolled and offered a corticosteroid injection. Patients were grouped based on whether they agreed to and received an injection or not. Range of motion (ROM) and patient-reported outcomes (PROs) were collected at the initial visit and Visual Analog Scale (VAS), Single Assessment Numeric Evaluation (SANE), and Disabilities of the Arm, Shoulder and Hand (DASH) questionnaire scores were collected using questionnaires emailed 2 weeks after the initial visit. This was a prospective, non-randomized observational study with treatment determined by patient preference. VAS and DASH were the primary outcomes. **Results:** 98 patients were enrolled and 73 patients received a corticosteroid injection. Follow-up PROs were completed by 47 injection patients and 18 non-injected patients. For injection patients, VAS scores improved from 5.3 ± 2.5 to 3.8 ± 2.8 (*p* < 0.001), and DASH scores improved from 42.2 ± 17.7 to 27.0 ± 21.7 (*p* < 0.001). Patients without injection also improved in VAS (5.1 ± 2.7 to 3.9 ± 2.7, *p* = 0.015) and DASH scores (38.3 ± 23.8 to 27.0 ± 18.8, *p* = 0.006). Between-group differences in changes in VAS, DASH, and affected-side SANE scores were not statistically significant (*p* = 0.661, *p* = 0.405, and *p* = 0.512, respectively). **Conclusions:** No statistically significant differences in pain or affected-shoulder function between groups were detected at approximately 2 weeks in this small, non-randomized study. These findings do not establish equivalence or determine whether clinical criteria identify patients who benefit from injection. Further studies should assess whether clinical and imaging findings improve patient selection.

## 1. Introduction

Subacromial bursitis of the shoulder is an inflammatory condition causing significant pain and reduced shoulder function for patients, often impairing activities of daily living and work productivity [1,2]. It is a common condition of the shoulder, and it is often accompanied by subacromial impingement syndrome [3,4]. Beyond its role in pain generation, the subacromial bursa is biologically active tissue, and bursa-derived cells are being investigated for biologic augmentation of rotator cuff repair [5]. There is level I evidence that treating subacromial bursitis with corticosteroid injections can be more effective than placebo injections or physiotherapy in reducing pain and improving shoulder function over short follow-up periods, depending on the population and comparator [6,7,8,9,10]. Physical therapy in combination with corticosteroid injection is an option for treating subacromial bursitis, with combined therapy not consistently demonstrating superior results to either physical therapy or injection alone [9,11]. Other conservative adjuncts, such as kinesio taping applied to the supraspinatus, have also been studied for their effects on shoulder strength and upper-extremity stability, although largely in healthy individuals [12]. However, current diagnostic criteria are not specific for subacromial bursitis, and identifying patients who will likely benefit from corticosteroid injection remains challenging [13].

Clinically suspected subacromial bursitis is diagnosed based on clinical history and physical examination findings, including anterolateral shoulder pain exacerbated by overhead activity and positive impingement tests, such as the Hawkins-Kennedy and Neer tests [13,14,15]. Additional physical examination tests such as the non-provocative arm traction abduction test continue to be proposed to improve the clinical diagnosis of subacromial impingement syndrome [16]. However, the clinical signs of subacromial bursitis greatly overlap with those of other shoulder pathologies, such as rotator cuff tendinopathy, rotator cuff tears and frozen shoulder [15,17,18]. Furthermore, diagnostic ultrasound to confirm the diagnosis, as performed in randomized controlled trials that demonstrate a clear benefit of corticosteroid injection, was not routinely employed in our practice [8,9,10,19]. The heterogeneity and limited specificity of physical examination findings in patients with clinically suspected subacromial bursitis make correctly identifying patients who may benefit from corticosteroid injections difficult [14,20]. Accordingly, further work is needed to establish criteria for when to perform an injection and a classification system for subacromial bursitis to guide clinical management [21,22]. This clinical approach was the reason for studying these patients.

Thus, this study compared the short-term outcomes of patients who met those criteria and received an injection with those who met the criteria and refused an injection, to describe outcomes following the currently employed clinical criteria for indicating corticosteroid injections in patients with symptoms of clinically suspected subacromial bursitis.

## 2. Methods

### 2.1. Study Overview

This was a prospective, non-randomized observational study. From July 2024 to April 2025, all patients who presented to a single fellowship-trained shoulder surgeon’s academic practice with signs and symptoms of clinically suspected subacromial bursitis were offered enrollment in the study. Each patient who met the clinical criteria for suspected subacromial bursitis was offered a corticosteroid injection. Range of motion (ROM) and patient-reported outcomes (PROs) were collected during a patient’s initial visit, and PROs were again collected using questionnaires emailed 2 weeks after the patient’s initial visit to obtain short-term outcomes. To compare outcomes after the same clinical indication, patients who received an injection were compared to those who were offered but refused an injection during their initial visit. Patients were excluded if they were under 18 years old, if they had prior shoulder surgery or trauma, or if study enrollment was refused. Treatment was determined by patient preference. No other treatment for the presenting shoulder symptoms occurred during follow-up in either group.

The study was approved by an Institutional Review Board (STUDY-23-01425; 28 March 2024). Written informed consent was obtained by a study investigator (CAS) before enrollment. The study was not prospectively registered.

### 2.2. Clinical Criteria for Suspected Subacromial Bursitis

Clinically suspected subacromial bursitis was diagnosed based on clinical criteria evaluated by a fellowship-trained orthopedic shoulder surgeon. Patients were diagnosed with clinically suspected subacromial bursitis during their visit if they demonstrated anterolateral shoulder pain in combination with a positive Hawkins-Kennedy test and a positive Neer test. These criteria provided a clinical working diagnosis; isolated bursal inflammation was not confirmed by ultrasound or MRI. Repeatability of the clinical tests and visual ROM estimates was not assessed. Measurement error may have affected eligibility and the composition of the cohort.

### 2.3. Exclusion Criteria

Patients were excluded if they demonstrated weakness in shoulder forward elevation, external rotation or internal rotation. Additional exclusion criteria were recent trauma, history of rotator cuff surgery, history of rotator cuff injury, loss of range of shoulder motion/stiffness, numbness, tingling, history of cervical pathology, glenohumeral arthritis on radiographs, acromioclavicular arthritis on radiographs, pain onset greater than 12 weeks before presentation and prior shoulder surgery.

### 2.4. Corticosteroid Injection

Patients diagnosed with clinically suspected subacromial bursitis were offered corticosteroid injection during their visit. Patients who agreed to receive an injection were injected with 80 mg of methylprednisolone acetate and 4 mL of 1% lidocaine without epinephrine into the subacromial space by the same fellowship-trained shoulder surgeon. The patient’s shoulder was exposed and prepped in standard fashion with chlorhexidine wipes. Using a posterolateral approach, a 22-gauge needle was introduced to the subacromial space. A trial drawback was performed to confirm no vascular structures had been violated and then the entirety of the corticosteroid mixture was injected into the subacromial space using the attached syringe. All injections were landmark-guided, and 80 mg was the treating surgeon’s usual protocol. The corticosteroid was supplied as an 80 mg/mL preparation, so the 80 mg dose corresponded to 1 mL, giving a total injected volume of 5 mL. Adverse events were not systematically solicited; they were recorded only if reported spontaneously by the patient or identified at a subsequent clinical encounter.

### 2.5. Clinical Evaluation and Outcome Measures

Clinical variables included forward elevation (FE), internal rotation (IR), external rotation (ER), visual analog scale (VAS), Disabilities of the Arm, Shoulder and Hand questionnaire (DASH) and the Single Assessment Numeric Evaluation (SANE) score. Active ROM and PROs were collected at the initial visit. VAS, SANE and DASH were also collected using an online questionnaire emailed 2 weeks after the initial visit. Most patients responded the same day or within the following one to two days. This follow-up timepoint was chosen to assess early symptomatic change following corticosteroid injection [8]. ROM was estimated visually by the same fellowship-trained orthopedic shoulder surgeon. ROM described baseline mobility and helped assess stiffness; it was not a follow-up outcome. VAS pain and DASH were the prespecified primary outcomes; contralateral SANE was exploratory. Higher VAS (0–10) and DASH (0–100) scores indicate worse symptoms, while higher SANE (0–100) scores indicate better function.

### 2.6. Statistical Analysis

All analyses were performed using Python version 3.8.8 (Python Software Foundation, Wilmington, DE, USA). Within-group comparisons between baseline and follow-up data were done via paired Student’s *t*-tests. Between-group comparisons of follow-up scores and changes from baseline were performed via unpaired *t*-tests. Statistical significance was defined as *p* < 0.05. These unadjusted analyses were exploratory; no adjustment for multiple comparisons was applied. Enrollment covered a fixed period, without an a priori sample-size calculation. Ninety-five per cent confidence intervals are reported for within-group mean changes and for between-group differences, calculated from the same *t*-test outputs. No adjustment was made for multiple comparisons.

## 3. Results

**Figure 1:**
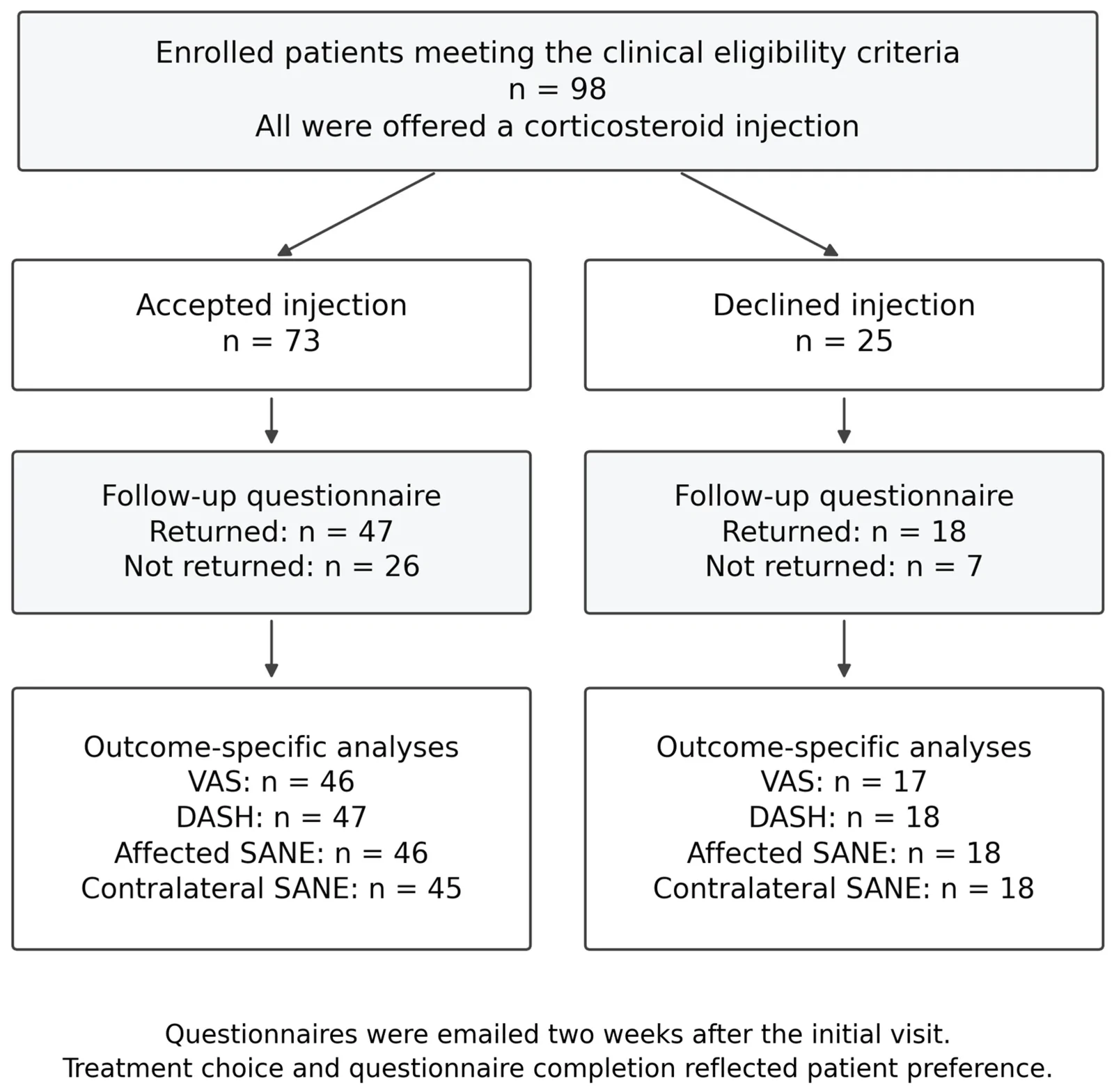
Participant flow, treatment choice and follow-up questionnaire completion. Numbers contributing to each outcome analysis are shown separately.

A total of 98 patients with clinically suspected subacromial bursitis were enrolled and assessed at baseline (49% female, median age 57 years [IQR: 42.25–65.75]; median BMI 26.4 kg/m^2^ [IQR: 23.90–30.00]; median follow-up 14 days [IQR: 14–16]). Of these, 73 patients received a subacromial corticosteroid injection, of whom 47 filled out patient-reported outcome questionnaires at follow-up (54.5% female, median age 58 years [IQR: 49–63], median BMI 25.8 kg/m^2^ [IQR: 24.8–28.5], median follow-up 15 days [IQR: 14–15]). 25 patients did not receive an injection, of whom 18 completed the follow-up questionnaire (43.8% female, median age 58 years [IQR: 38–65], median BMI 26.8 kg/m^2^ [IQR: 21.8–32.8], median follow-up 14 days [IQR: 14–17]). Participant flow is shown in Figure 1; 26 and 7 patients, respectively, did not return the questionnaire. Questionnaire completion was voluntary. No adverse events were reported.

### 3.1. Baseline Range of Motion

Baseline shoulder range of motion was recorded at the initial visit (Table 1, Panel B). The mean forward elevation was 174 ± 15 degrees, and the mean external rotation was 55 ± 13 degrees (*n* = 98). Internal rotation, measured by vertebral level, had a median of L1 (IQR: L3 to T12) (n = 96). Distributions for forward elevation and external rotation are illustrated in Supplementary Figures S1 and S2, respectively. Baseline forward elevation clustered near 180 degrees, indicating a ceiling effect; change in ROM was not assessed at follow-up.

### 3.2. Within-Group Improvements in Patient-Reported Outcomes

At follow-up, patients who received a corticosteroid injection reported significant improvements in pain and function. VAS scores improved from 5.3 ± 2.5 to 3.8 ± 2.8 (*p* < 0.001), and DASH scores improved from 42.2 ± 17.7 to 27.0 ± 21.7 (*p* < 0.001). The SANE score for the affected shoulder showed a non-significant increase from 55.7 ± 22.7 to 63.3 ± 28.2 (*p* = 0.089), while scores for the non-affected side did not change significantly (92.1 ± 12.5 to 92.3 ± 17.1, *p* = 0.938) (Table 2). Mean changes with 95% confidence intervals were −¼ points for VAS (−2.1 to −0.8). −15.2 points for DASh (−20.5 to −10.0). 7.6 points for affected side SANE (−1.2 to 16.4) and 0/2 points for the non-affected side (−3.8–4.1).

Patients who did not receive an injection also demonstrated statistically significant improvements in VAS (5.1 ± 2.7 to 3.9 ± 2.7, *p* = 0.015) and DASH scores (38.3 ± 23.8 to 27.0 ± 18.8, *p* = 0.006). Changes in SANE scores were not statistically significant for either the affected (58.3 ± 24.9 to 60.6 ± 25.5, *p* = 0.744) or non-affected side (93.9 ± 14.5 to 81.1 ± 33.7, *p* = 0.133) (Table 3). Mean changes with 95% confidence intervals were −1.2 points for VAS (−2.1 to −0.3), −11.3 points for DASH (−18.8 to −3.7), 2.2 points for affected-side SANE (−11.9 to 16.3) and −12.8 points for the non-affected side (−29.9 to 4.3).

### 3.3. Comparison of Outcomes by Treatment Group

At follow-up, there were no statistically significant differences in patient-reported outcome scores between the injection and non-injection groups (Table 4). VAS scores did not differ significantly (3.8 ± 2.8 vs. 3.9 ± 2.7, *p* = 0.965), nor did DASH scores (27.0 ± 21.7 vs. 27.0 ± 18.8, *p* = 0.995). There were no significant between-group differences in the change in VAS (*p* = 0.661), DASH (*p* = 0.405), or SANE affected-side scores (*p* = 0.512). The only significant difference observed was in the change in SANE scores for the non-affected shoulder, which declined in the non-injection group (−12.8 ± 34.4) but remained stable in the injection group (0.2 ± 13.3; *p* = 0.034). This contralateral finding was exploratory and may reflect chance or measurement variability among multiple comparisons. Between-group differences and their 95% confidence intervals are given in Table 4. For the change in VAS the difference was −0.3 points (95% CI −1.4 to 0.9), for DASH −4.0 points (95% CI −13.5 to 5.5) and for affected-side SANE 5.4 points (95% CI −10.9 to 21.7). These intervals remain compatible with differences that some patients would consider worthwhile, so the absence of a statistically significant difference should not be read as evidence of equivalence.

## 4. Discussion

Subacromial bursitis is a common condition of the shoulder often causing severe pain and disability for patients, and corticosteroid injections have been shown to be an effective short-term treatment in randomized trials using ultrasound imaging to confirm the diagnosis. However, the indication for corticosteroid injection is often made based on rather nonspecific clinical examination findings without the use of diagnostic imaging [19]. Thus, this study compared the short-term outcomes of patients with clinical signs of suspected subacromial bursitis who received an injection with those who were offered an injection but refused it. The clinical criteria used to indicate subacromial bursa corticosteroid injection were anterolateral shoulder pain in combination with positive Hawkins-Kennedy and Neer tests [9,11,23]. No diagnostic ultrasound was used to confirm the diagnosis, reflecting the approach used in our practice [19]. Each patient who fulfilled those clinical criteria was offered an injection, and we found no statistically significant difference in patient-reported outcomes at approximately 2 weeks between patients with and without an injection, and the VAS and DASH scores improved significantly in both groups between baseline and 2 weeks. As patients in both groups had a similar indication for corticosteroid injection, these findings describe outcomes after clinical selection, but do not establish whether the clinical criteria identify patients who benefit from injection.

Recent level I evidence has demonstrated short-term benefits of corticosteroid injection for subacromial bursitis compared with dextrose, physiotherapy, or saline under the studied conditions [8,9,10]. It is important to note that these studies used ultrasound imaging in addition to clinical examination findings to confirm the presence of subacromial bursitis. Our results do not establish the ineffectiveness of corticosteroid injection as a treatment. Our study examines the current approach to diagnosing clinically suspected subacromial bursitis in our practice, without ultrasound confirmation [19]. British Elbow and Shoulder Society/British Orthopaedic Association guidance for primary, community and intermediate care bases diagnosis on history and examination and does not recommend routine ultrasound or MRI in these settings [24]. That guidance applies to primary, community and intermediate care rather than to specialist practice, but it illustrates that a diagnosis based on history and examination remains standard in settings where imaging is not routinely available.

Minimal clinically important differences reported in frozen shoulder and shoulder arthroplasty populations should not be directly applied to this cohort [25,26,27]. In the corticosteroid injection group, DASH improved by 15.2 points. Mean VAS improved by 1.4 points over the same interval. In the group that did not receive an injection the corresponding changes were 1.2 points for VAS and 11.3 points for DASH, and none of the between-group differences reached statistical significance (Table 4). The corresponding between-group differences were −0.3 points for VAS (95% CI −1.4 to 0.9) and −4.0 points for DASH (95% CI −13.5 to 5.5). Because these intervals span values that would be considered meaningful in either direction, our data neither demonstrate a benefit of injection nor exclude a benefit.

Another feature of this study is the use of a similar corticosteroid dose for each patient [28,29]. Neither the degree of pain and functional impairment nor a patient’s weight or age were used to adjust the dose in this cohort; 80 mg was the treating surgeon’s usual protocol. This study did not compare doses, nor is it known whether adjusting dosages for each individual patient and their symptom severity would be beneficial.

Ultrasound imaging is often used to diagnose subacromial bursitis in a dichotomous fashion (“bursitis” versus “no bursitis”), and Lee et al. reported that patients with signs of bursitis on ultrasound, such as bursa thickening and effusions, had greater improvement in some outcomes after corticosteroid injection than those without those findings [30]. However, further work is needed to develop classification systems for subacromial bursitis, whether based on ultrasonography or clinical findings, that could be used to guide management, including the indication of corticosteroid injection and appropriate dosing. Accordingly, future prospective studies are needed to identify specific degrees of subacromial bursitis and to guide management based on the severity of both clinical and imaging findings to provide a more patient-centered and evidence-based approach to treating subacromial bursitis. A cohort with ultrasound-confirmed bursitis and randomized treatment allocation may help address these questions.

## 5. Limitations

In this study, patients were enrolled during their regular office visits and each patient who met clinical criteria for suspected subacromial bursitis was offered corticosteroid injection. The no-injection group included patients who were offered but refused an injection. This self-selection inherently created a selection bias and differently sized study groups, with more people agreeing to receive an injection than not. Treatment expectations and symptom trajectories may have differed, including improvement before the visit among patients who declined injection. The unadjusted analyses do not account for these differences. Nonetheless, we believe that the statistical analysis used overall supports the conclusions drawn in this study. Another source of selection bias was the use of a voluntary online questionnaire to obtain PROs at 2 weeks, and the direction of bias from missing follow-up is uncertain. Furthermore, while pain and shoulder function are expected to improve within two weeks from corticosteroid injection, it is possible that a longer follow-up duration would have shown different results when comparing both groups [8]. The small comparison group limits precision, and nonsignificant results do not establish equivalence or exclude a clinically meaningful difference. The isolated significant SANE non-affected-side finding should be interpreted in the context of multiple statistical comparisons. Injection placement was not confirmed by ultrasound. Injection was offered to every patient who met the clinical criteria as part of a routine clinical encounter; no standardized counseling script was used, so the framing may have varied between encounters. Comorbidities relevant to corticosteroid use, including diabetes, were not recorded, and their distribution between the groups is therefore unknown. Adverse events were captured by spontaneous report only, so unreported events cannot be excluded. The confidence intervals around the between-group differences quantify this imprecision and are reported in Table 4.

Despite these limitations, we believe that this study’s design and its results reflect the real-life approach to diagnosing and managing clinically suspected subacromial bursitis in our practice, [24] and provide a basis for further studies of clinical criteria used to select patients for injection.

## 6. Conclusions

Current clinical criteria for diagnosing clinically suspected subacromial bursitis rely heavily on physical examination findings that overlap with other shoulder conditions. In this study, patients who were offered and who received a corticosteroid injection and those who were offered but declined an injection demonstrated no statistically significant difference in improvement in pain or affected-shoulder function at approximately 2 weeks after their initial visit. However, this small, non-randomized comparison does not establish equivalence or determine whether the clinical criteria identify patients who benefit from injection. Future investigations may therefore focus on the imaging-based diagnosis, identifying factors that could dictate steroid dosing and creating classification systems that could more definitively identify patients who may benefit from corticosteroid injection to enhance outcomes and optimize care for subacromial bursitis patients.

## Figures and Tables

**Table 1:** Baseline characteristics and range of motion at initial visit for patients with clinically suspected bursitis. Presented as mean (SD), mean ± SD, median and interquartile range (IQR), or percentage. SD, standard deviation. Sample sizes for patient-reported outcomes are given by row.

**Panel A. Group-Specific Baseline Characteristics.**		
**Characteristic**	**Injection**	**No Injection**
Age, years; median (IQR)	58 (49 to 63)	58 (38 to 65)
Female sex, %	54.5	43.8
BMI, kg/m^2^; median (IQR)	25.8 (24.8 to 28.5)	26.8 (21.8 to 32.8)
VAS, mean (SD)	5.3 (2.5); n = 46	5.1 (2.7); n = 17
DASH, mean (SD)	42.2 (17.7); n = 47	38.3 (23.8); n = 18
SANE, affected shoulder; mean (SD)	55.7 (22.7); n = 46	58.3 (24.9); n = 18
SANE, non-affected shoulder; mean (SD)	92.1 (12.5); n = 45	93.9 (14.5); n = 18
**Panel B. Baseline Range of Motion.**		
Forward Elevation (n = 98), Mean ± SD	174 ± 15	
External Rotation (n = 98), Mean ± SD	55 ± 13	
Internal Rotation (n = 96), Median (IQR)	L1 (L3 to T12)	

**Table 2:** Patient-reported outcomes at initial visit and approximately two weeks later for patients **who received an injection.** Values are mean (SD); n is the number contributing to each comparison. SD, standard deviation; VAS, visual analog scale; DASH, Disabilities of the Arm, Shoulder and Hand; SANE, Single Assessment Numeric Evaluation. Mean change is follow-up minus baseline, shown with its 95% confidence interval from the paired *t*-test. CI, confidence interval.

	Baseline	2-Week Follow-Up	Mean Change (95% CI)	*p*-Value
VAS (n = 46)	5.3 (2.5)	3.8 (2.8)	−1.4 (−2.1 to −0.8)	**<0.001**
DASH (n = 47)	42.2 (17.7)	27.0 (21.7)	−15.2 (−20.5 to −10.0)	**<0.001**
SANE Affected Side (n = 46)	55.7 (22.7)	63.3 (28.2)	7.6 (−1.2 to 16.4)	0.089
SANE Non-Affected Side (n = 45)	92.1 (12.5)	92.3 (17.1)	0.2 (−3.8 to 4.1)	0.938

**Table 3:** Patient-reported outcomes at initial visit and approximately two weeks later for **patients who did not receive an injection.** Values are mean (SD); n is the number contributing to each comparison. SD, standard deviation; VAS, visual analog scale; DASH, Disabilities of the Arm, Shoulder and Hand; SANE, Single Assessment Numeric Evaluation. Mean change is follow-up minus baseline, shown with its 95% confidence interval from the paired *t*-test. CI, confidence interval.

	Baseline	2-Week Follow-Up	Mean Change (95% CI)	*p*-Value
VAS (n = 17)	5.1 (2.7)	3.9 (2.7)	−1.2 (−2.1 to −0.3)	**0.015**
DASH (n = 18)	38.3 (23.8)	27.0 (18.8)	−11.3 (−18.8 to −3.7)	**0.006**
SANE Affected Side (n = 18)	58.3 (24.9)	60.6 (25.5)	2.2 (−11.9 to 16.3)	0.744
SANE Non-Affected Side (n = 18)	93.9 (14.5)	81.1 (33.7)	−12.8 (−29.9 to 4.3)	0.133

**Table 4:** Final 2-week and interval change in patient-reported outcomes between patients who did and did not receive an injection after a clinical diagnosis of suspected bursitis. Values are mean (SD); changes are follow-up minus baseline. The two n values refer to the injection and non-injection groups, respectively. SD, standard deviation; VAS, visual analog scale; DASH, Disabilities of the Arm, Shoulder and Hand; SANE, Single Assessment Numeric Evaluation. Differences are the injection group minus the non-injection group, shown with 95% confidence intervals from the unpaired *t*-test assuming equal variances. CI, confidence interval.

	Injection	No Injection	Between Group Difference (95% CI)	*p*-Value
2-Week VAS (n = 46, 17)	3.8 (2.8)	3.9 (2.7)	0.0 (−1.6 to 1.5)	0.965
2-Week DASH (n = 47, 18)	27.0 (21.7)	27.0 (18.8)	0.0 (−11.6 to 11.6)	0.995
2-Week SANE Affected Side (n = 46, 18)	63.3 (28.2)	60.6 (25.5)	2.7 (−12.6 to 18.0)	0.724
2-Week SANE Non-Affected Side (n = 45, 18)	92.3 (17.1)	81.1 (33.7)	11.2 (−1.7 to 24.0)	0.087
VAS Change (n = 46, 17)	−1.4 (2.2)	−1.2 (1.8)	−0.3 (−1.4 to 0.9)	0.661
DASH Change (n = 47, 18)	−15.2 (17.8)	−11.3 (15.3)	−4.0 (−13.5 to 5.5)	0.405
SANE Affected Side Change (n = 46, 18)	7.6 (29.7)	2.2 (28.3)	5.4 (−10.9 to 21.7)	0.512
SANE Non-Affected Side Change (n = 45, 18)	0.2 (13.3)	−12.8 (34.4)	12.9 (1.0 to 24.9)	**0.034**

## Data Availability

The datasets presented in this article are not readily available because the data are part of an ongoing study. Requests to access the datasets should be directed to Christoph.schroen@googlemail.com.

## Supplementary Materials

The following supporting information can be downloaded at https://www.mdpi.com/article/10.3390/jcm15207863/s1. Figure S1: Distribution of baseline forward elevation in patients with clinical findings suggestive of subacromial bursitis. Figure S2: Distribution of baseline external rotation in patients with clinical findings suggestive of subacromial bursitis.
