# Supplementary material for: Short-Term Outcomes After Corticosteroid Injection in Patients with Clinically Diagnosed Subacromial Pain Suggestive of Bursitis
Source: J Clin Med. 2026 Oct 12;15(20):7863. doi: 10.3390/jcm15207863 (PMC13705209; doi:10.3390/jcm15207863)
Supplement: Supplementary file 1 [file jcm-15-07863-s001.zip › jcm-4512881-supplementary.pdf]

Supplementary Figure S1

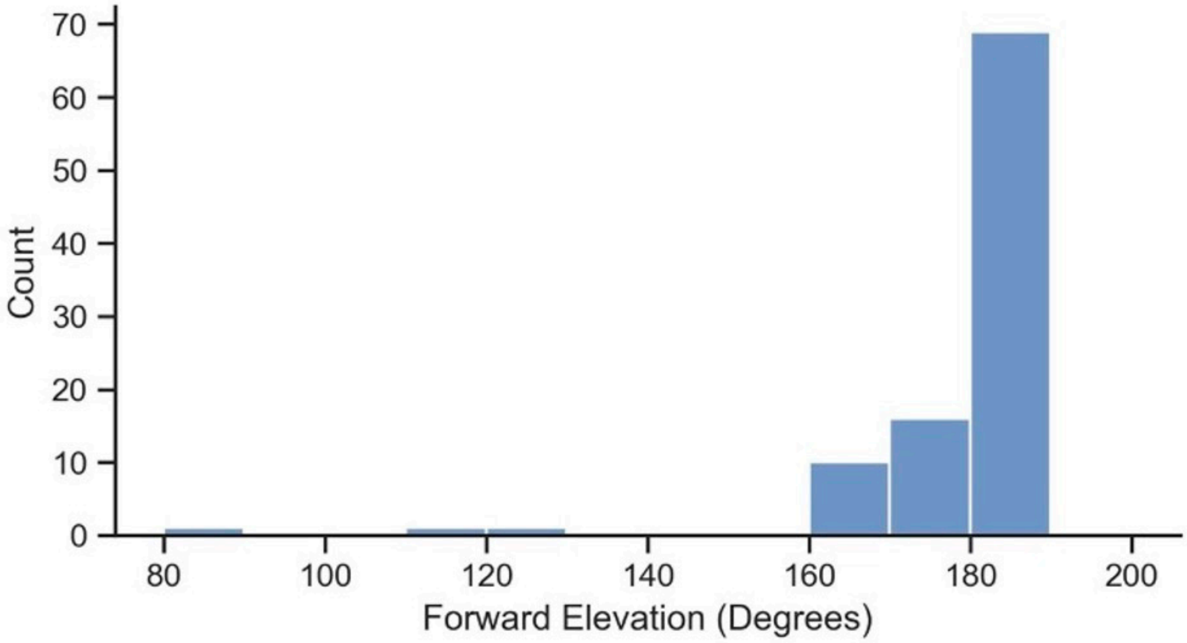

Distribution of baseline forward elevation in patients with clinical findings suggestive of subacromial bursitis.

Supplementary Figure S2

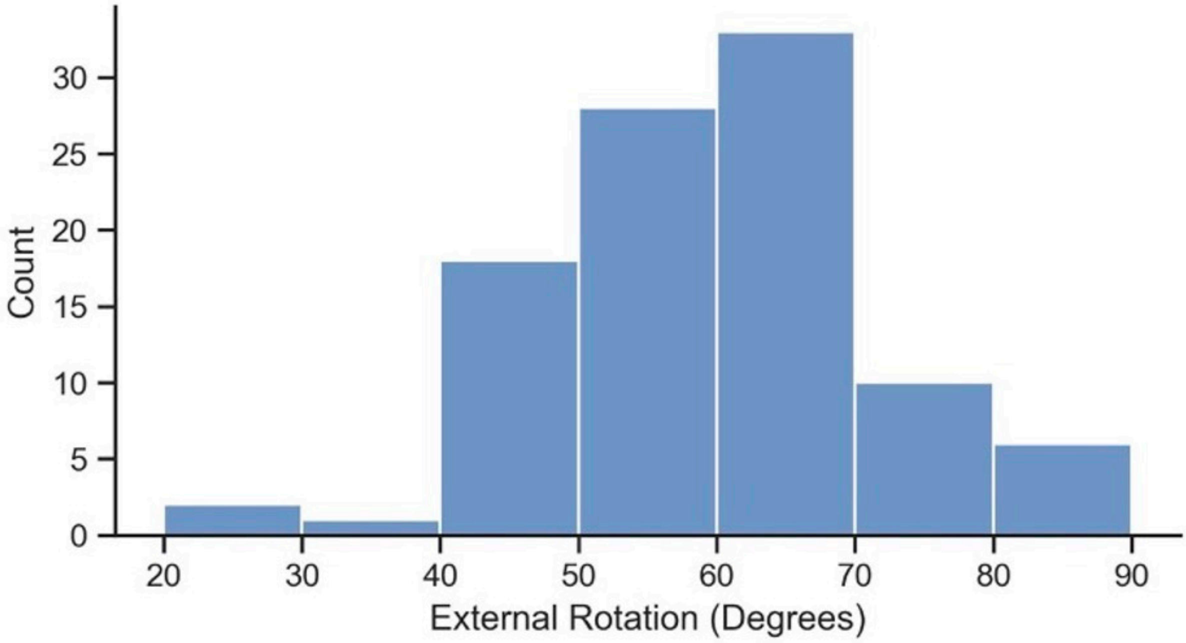

Distribution of baseline external rotation in patients with clinical findings suggestive of subacromial bursitis.
